# RNA-Seq-Based Gene Expression Pattern and Morphological Alterations in Chick Thymus during Postnatal Development

**DOI:** 10.1155/2019/6905194

**Published:** 2019-04-21

**Authors:** Zhouyiyuan Xue, Abdur Rahman Ansari, Xing Zhao, Kun Zang, Yu Liang, Lei Cui, Yafang Hu, Ranran Cheng, Xiaolong Zhang, Juming Zhong, Huazhen Liu

**Affiliations:** ^1^Department of Basic Veterinary Medicine, College of Animal Science and Veterinary Medicine, Huazhong Agricultural University, Wuhan, Hubei 430070, China; ^2^Section of Anatomy and Histology, Department of Basic Sciences, College of Veterinary and Animal Sciences (CVAS) Jhang, University of Veterinary and Animal Sciences (UVAS), Lahore, Pakistan; ^3^Department of Anatomy, Physiology and Pharmacology, College of Veterinary Medicine, Auburn University, Auburn, USA

## Abstract

The thymus is a lobulated unique lymphoid immune organ that plays a critical role in the selection, development, proliferation, and differentiation of T cells. The thymus of developing chickens undergoes continued morphological alterations; however, the biomolecular and transcriptional dynamics of the postnatal thymus in avian species is not clear yet. Therefore, the thymuses from chickens at different stages of development (at weeks 0, 1, 5, 9, 18, and 27) were used in the present study. The RNA-seq method was used to study the gene expression patterns. On average, 24120819 clean reads were mapped, differentially expressed genes (DEGs) were identified on the basis of log values (fold change), including 744 upregulated and 425 downregulated genes. The expression pattern revealed by RNA-seq was validated by quantitative real-time PCR (qPCR) analysis of four important genes, which are PCNA, CCNA2, CCNB2, and CDK1. Thus, the current study revealed that during postnatal development, the thymus undergoes severe atrophy. Thymus structure was damaged and gene expression changed dramatically, especially at the 27^th^ week of age. Moreover, we found significant changes of several signaling pathways such as the cytokine-cytokine receptor interaction and cell cycle signaling pathways. Hence, it may be inferred that those signaling pathways might be closely related to the postnatal chicken thymus development.

## 1. Introduction

The thymus develops as a gland and is considered as a lobulated unique lymphoid organ [1, 2]. As a primary immune organ, it acts as the main player in the selection, development, proliferation, and differentiation of T cells [3, 4]. Although it has prime importance in immune functions [5, 6], it experiences a conserved evolutionary process of shrinkage with aging in almost all vertebrates that causes a decrease in thymic tissue mass as well as alters its architecture [7].

The development of T cells begins with the migration of multipotent progenitors from the bone marrow to the thymus [8]. The complicated microenvironment in the thymus provides the site for naïve T cells to mature under a specific cascade of transcriptional factors [9]. As a primary immune organ, it plays a crucial role in acquired immunity with the expression of several intrathymic transcripts [10]. The thymus morphology changes with age, and its cellular components may also play an important role in prenatal immunity [11]. Aging is described as the gradual loss of physiology and cellular morphology based on cell-to-cell transcriptional variability in many mammalian tissues [12]. Studies showed that with the increase in age, the thymus undergoes a severe involution, manifesting as increased cell death and fibrosis, concomitant with a loss of T cell production [13–15]. But the molecular mechanism and when this process initiates remain undetermined [16]. Since thymic morphology and cell types are evolutionarily preserved in several vertebrate species [17, 18], the thymus might have similar age-related changes in chickens [18].

The transcriptional signature of age-related thymic involution shows altered expression of certain genes and transcriptional factors [16]. The development of the thymus is regulated by many transcriptional factors such as the thymic epithelial cell- (TEC-) specific transcription factor Forkhead box N1 (*Foxn1*), transcription factor *Fezf2*, and thymic stromal lymphopoietin (*TSLP*)/*TSLP* Receptor (*TSLPR*) through several signaling pathways in mammalian species [19–21]. Although recent studies have provided much information about several key genes, signaling pathways functioning during thymus postnatal development need further studies. Furthermore, intrinsic mediators responsible for biological pressure and mechanisms of decreased thymic function as well as the molecular and transcriptional factors of thymus development and its atrophy are still unclear in avian species. Therefore, in the current study, we conducted RNA-seq to provide a macroscopic picture of the gene expression and explored the mechanism on how aging affects transcriptional dynamics using RNA sequencing of thymuses from developing chicks without immunological activation.

## 2. Materials and Methods

### 2.1. Ethics Statement

The current scientific investigation was conducted in accordance with the rules and regulations of the ethics committee for use of animals, Huazhong Agricultural University (HZAU), Wuhan, China.

### 2.2. Animals

One-day-old, healthy Cobb500 strain broiler chicks were purchased from the Wuhan Zhengda chicken breeding company. The newly hatched chicks were reared under conventional housing conditions. For transcriptome analysis of the chick thymus during aging, three chicks per time point (0, 1, 5, 9, 18, and 27 weeks) were sacrificed. Thymuses were harvested from each bird and weighed at certain defined time points. The thymus index was calculated using the following formula: thymus index (g/kg) = weight of thymus (g)/body weight (kg) [22, 23]. For histomorphological analysis, parts of freshly harvested thymuses were fixed in 4% paraformaldehyde solution, embedded into paraffin wax, and stored at 4°C. For molecular studies and gene expression analysis, parts of freshly harvested thymuses were snap frozen in liquid nitrogen and stored at -80°C.

### 2.3. Hematoxylin and Eosin Staining

The paraffin wax thymic tissue blocks were cut into 4 *μ*m thick tissue sections. After deparaffinization in xylene, the tissue sections were rehydrated in a graded series of alcohol to water. Staining of tissue sections was performed with Harris hematoxylin solution for 5 min and then differentiated in acid alcohol for 10 s. The tissue sections were rinsed under running tap water for 15 min and subsequently stained in eosin solution for 1 min. After dehydration in alcohol, the tissue sections were cleared in xylene and finally mounted with glass coverslips. Stained tissue sections were thoroughly examined by light microscopy (Olympus BX51, Tokyo, Japan) with an attached digital camera (DP72, Olympus).

### 2.4. RNA-Seq and Data Analysis

Tissue specimens were sent to BGI Genomics Co. Ltd. to conduct transcriptome sequencing. In this study, different time points were compared with the 0 week to identify DGEs. Three biological replicates were studied for each time point. Following construction of strand-specific libraries, sequencing was done on an Illumina HiSeq 4000 platform by BGI Genomics Co. Ltd. (Shenzhen, China). Differentially expressed genes (DEGs) were screened using the NOISeq method with absolute fold change ≥ 2 and divergent probability ≥ 0.8 [24, 25]. The read length was 50 bp. Perl was used for handling of raw data, and the quality of the obtained data was carefully checked with FastQC 0.11.2 (http://www.bioinformatics.babraham.ac.uk/projects/fastqc/). Clean reads in this study were aligned to the specific chicken genome database (release: Gallus gallus 4.0) from NCBI using HISAT, by allowing one mismatch. The annotation of genome mapped data was done using the GFF3 file of the Gallus gallus 4.0 genome (ftp://ftp.ncbi.nlm.nih.gov/genomes/Gallus_gallus/). The expression of the transcript was represented in terms of FPKM (fragments per kilobase of exon per million fragments mapped). Gene expression profile analysis, GO analysis (http://geneontology.org/page/go-enrichment-analysis), and KEGG analysis were conducted depending on the data. In this study, the significantly enriched signaling pathways were sorted out when *P* < 0.05.

### 2.5. RNA-Seq Data Validation by Quantitative Real-Time PCR (qPCR)

For the validation of RNA-seq data, total RNA from thymic tissue was isolated and the genomic DNA was then removed by treating with RNase-free DNase I (Fermentas, St. Leon-Rot, Germany). Synthesis of first strand cDNA was accomplished using a RevertAid First Strand cDNA Synthesis Kit (Fermentas, St. Leon-Rot, Germany). The total volume (10 *μ*L) of the qPCR reaction mixture contained 2 *μ*L of each forward and reverse primer, 1 *μ*L of template cDNA, and 5 *μ*L of SYBR Select Master Mix for CFX (Applied Biosystems, Inc., USA). The qPCR reaction was accomplished by the following conditions: predenaturation at 95°C (5 min), followed by denaturation (40 cycles) at 95°C (30 s), annealing at 60°C (30 s), and finally elongation at 72°C (20 s) The reference gene for qPCR was *β*-actin. All the primer sequences used in this study are listed in Table 1. All the thymic tissue RNA samples were run in triplicate, and the expression levels of all desired genes were calculated by the ΔΔCt method [26].

### 2.6. Statistical Analysis

Statistical data were calculated as the means ± standard deviation (SD). Statistical calculations and analyses as well as graphical representations were done with Prism software version 5.01 (GraphPad Software Inc., San Diego, USA). The statistical significance was accomplished with Bonferroni's multiple comparison test after a one-way ANOVA test.

## 3. Results

### 3.1. The Changes in Thymus Weight, Index, and Morphology during Postnatal Development

**Figure 1:**
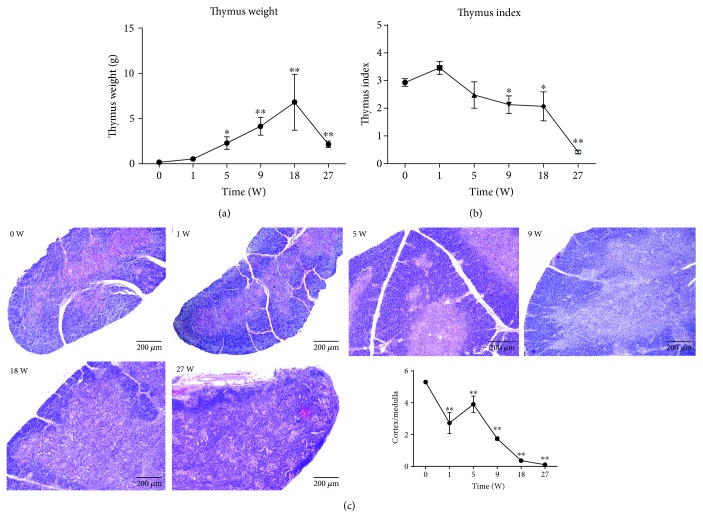
The changes of the thymus weight, index, and morphology during postnatal development. (a) The thymus weight kept increasing until the 18^th^ week and then decreased. (b) The thymus index continued to decline. (c) Sections of thymuses were stained with hematoxylin and eosin (H&E) staining to analyze changes of the tissue structure. Light areas represent the medulla, and dark areas represent the cortex. The cortex was thick, and the boundary between the cortex and medulla was clear at the 0 week. With the increase in age, the cortex became thinner and the boundary blurred at the 5^th^ week and was hardly distinguishable at the 27^th^ week. In addition, many round-shaped bubbles were formed due to the dissolved adipose tissue, which appeared at the 5^th^ week and kept increasing with the advancement of age. Scale bars = 200 *μ*m. All the data are presented as means ± SD. ^∗^ *P* < 0.05, ^∗∗^ *P* < 0.01.

The statistical analysis of chicken thymus weight and thymus index revealed that the thymus weight kept increasing with the increase of age and peaked at the 18^th^ week and then decreased (Figure 1(a)), while the thymus index increased at the first week and then decreased (Figure 1(b)). HE staining showed that the chicken thymus was covered by a capsule which penetrated into the thymus and divided it into many niches. Each niche contained an outer cortex and an inner medulla. The thickness of the cortex decreased, and the clear corticomedullary junction became blurred with the advancement in age of birds from 0 to the 5^th^ week and was extremely fuzzy at the 27^th^ week. Furthermore, several round-shaped bubbles of dissolved adipose tissue appeared at the 5^th^ week, and their number continuously increased with the postnatal growth of the birds (Figure 1(c)).

### 3.2. Identification of DEGs in Chicken Thymus during Postnatal Development

**Figure 2:**
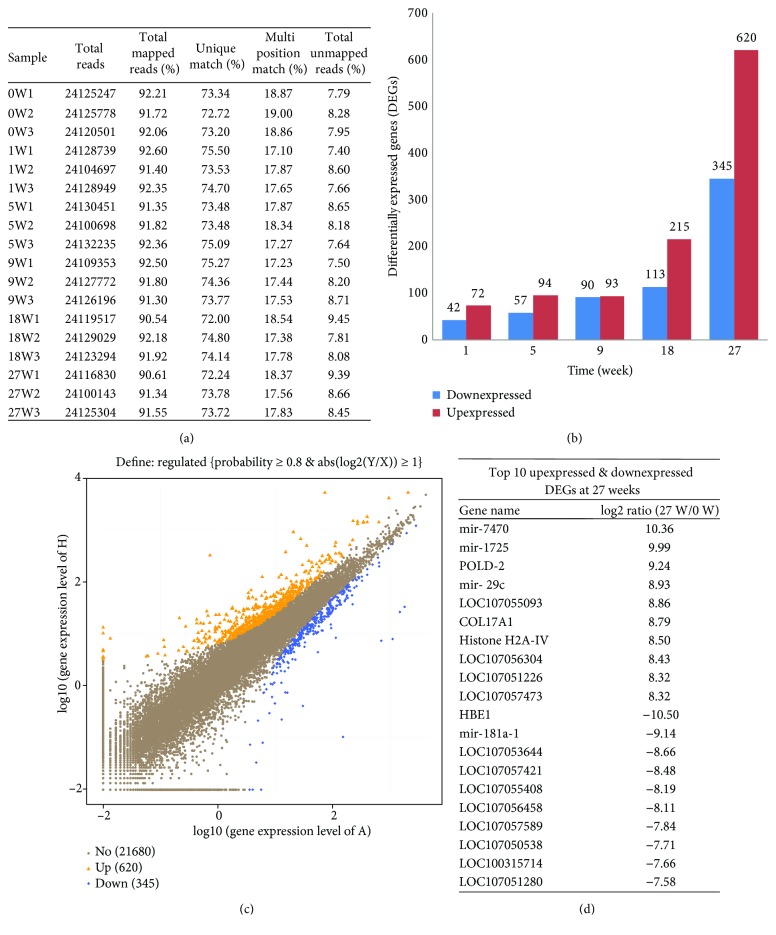
Analysis and validation of DEGs in the chicken thymus at different time points. (a) Alignment statistics of reads align to the reference genome. (b) The diagram shows total numbers of differentially expressed genes (DEGs) at 0 (1), 5, 9, 18, and 27 weeks. (c) Scatter plot diagram showing the log value of the gene expression of the 0-week thymus (*X*-axis) versus the log value of the gene expression of the 27^th^-week thymus (*Y*-axis). The blue color indicates downregulated genes, the orange color represents upregulated genes, and the brown color designates unchanged genes. Top legends on each figure show the statistics of screening threshold values. (d) Top 10 upregulated and downregulated DEGs at the 27^th^-week thymus. The positive numbers represented upregulated log values while negative numbers represent downregulated log values of DEGs.

The RNA-seq statistics pertaining to the average total reads, mapping reads, and unique matching for each of the 18 samples are shown in Figure 2(a). The differentially expressed genes (DEGs) were analyzed by using NOISeq software. Overall, there were 425 downregulated and 744 upregulated genes at all the five time points (0, 1, 5, 9, 18, and 27 weeks) (Supplementary File 1). Among these, we observed 114 DEGs (42 downregulated and 72 upregulated) at the 1^st^ week, 151 DEGs (57 downregulated and 94 upregulated) at the 5^th^ week, 183 DEGs (90 downregulated and 93 upregulated) at the 9^th^ week, 328 DEGs (113 downregulated and 215 upregulated) at the 18^th^ week, and 965 DEGs (345 downregulated and 620 upregulated) at the 27^th^ week (Figure 2(b)). Maximum DEGs were observed at the 27^th^ week; therefore, we selected the 27^th^ week as a key time point to conduct further analysis. The scatter plots of all the expressed genes were drawn to represent the distribution of DEGs in screening threshold dimensions (Figure 2(c)). The top ten upregulated and downregulated DEGs at the 27^th^ week are shown in Figure 2(d). Interestingly, the most of them are ncRNA (uncharacterized LOC *Gallus gallus* gene) and microRNA such as mir-7470, mir-1725, mir-29c, and mir-181a-1.

### 3.3. Bioinformatics Analysis of DEGs

**Figure 3:**
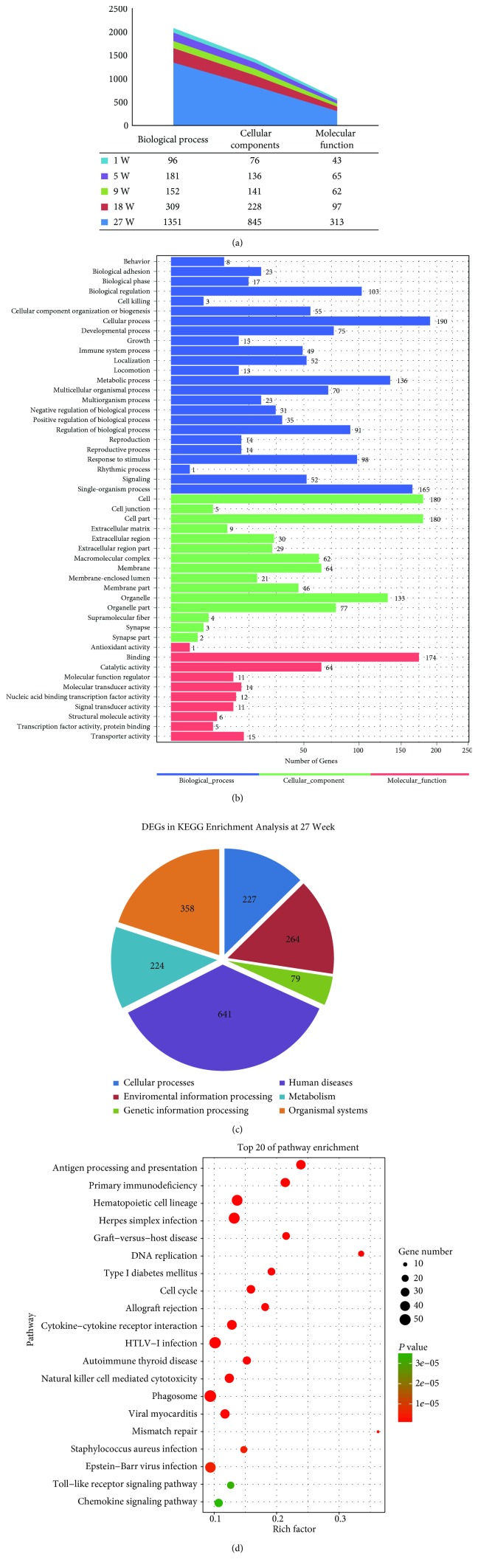
(a) Gene ontology (GO) analysis of DEGs at each time point showing enrichment of different commonly expressed genes in GO terms. (b) Diagram showing GO terms and their designated number of DEGs at the 27^th^-week thymus. The *X*-axis shows the number of DEGs while the *Y*-axis represents the GO terms. Three different colors designate three ontologies of GO terms: the blue color represents the biological process, the green color indicates cellular components, and the red color shows molecular functions. (c) Diagram showing the KEGG enrichment analysis of DEGs at the 27^th^-week thymus. (d) The *X*-axis shows the rich factor while the *Y*-axis represents different pathways. The colors represent the *P* value. The size of the dot represents the DEG number in a certain pathway.

GO functional classification on DEGs for each pair was conducted following the previously described method [25]. All GO terms are grouped into three ontologies: biological process, cellular component, and molecular function. We found that most DEGs were enriched in GO terms at the 27^th^ week, i.e., 1351 genes were annotated in biological processes, 845 genes in cellular components, and 313 genes in molecular function (Figure 3(a)). The top three enriched biological processes were the cellular process (190 genes), single-organism process (165 genes), and metabolic process (136 genes) (Figure 3(b)). The top three enriched cellular components were the cell (180 genes), cell part (180 genes), and organelle (133 genes). The top three enriched molecular function categories were the binding activity (174 genes), catalytic activity (64 genes), and transporter activity (15 genes) (Figure 3(b)). KEGG enrichment analysis of DEGs at the 27^th^ week showed enrichment of 227 DEGs in cellular processes, 264 in the environmental information system, 79 in genetic information processing, 641 in human diseases, 224 in the metabolism, and 358 in organismal systems related to the signaling pathways (Figure 3(c)). Further analysis showed that DNA replication, cell cycle, cytokine-cytokine receptor, and mismatch repair pathways were significantly enriched (Figure 3(d)).

### 3.4. RNA-Seq Data Validation by Quantitative Real-Time PCR (qPCR)

**Figure 4:**
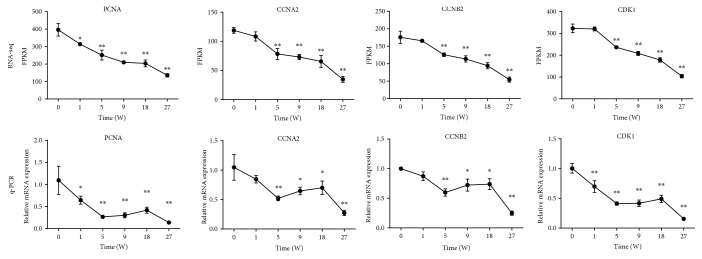
The expression patterns of four genes in chick thymuses were detected by qPCR. In order to test the reliability of the RNA-seq result, comparison analysis was performed for expression patterns of PCNA, CCNA2, CCNB2, and CDK1 genes.

The cell cycle signaling pathway is closely associated with cell proliferation and death; therefore, it may play an important role in postnatal development. PCNA, CCNA2, CCNB2, and CDK1 were chosen to conduct q-PCR. The expression patterns of these genes were similar to the results obtained from RNA-seq (Figure 4).

## 4. Discussion

Gene expression can be measured by the application of the next generation sequencing (NGS) technologies such as RNA sequencing (RNA-seq) [27–29], since the changes in gene expression may play an important role in postnatal development of the thymus [30, 31]. For better understanding of the gene expression pattern, we conducted transcriptome analysis of the thymus at different developmental stages. A large body of scientific research has also described the basic histogenetical rule that the postnatal vertebrate thymus experiences distinct morphological and functional alterations [7, 32, 33]. Morphologically, the weight of the immune organ reflects its developmental speed [34], while the index represents its maturity [35]. Maximum DEGs were observed at the 27^th^ week that dramatically coincided with alterations in the morphological structure of the thymus at the same time point as compared to the 0 week. In the current study, the increase in thymus weight before the 18^th^ week might be the result of the increase in cell number and cell volume in the early postnatal developmental stage while the decline at the 27^th^ week may be the result of the age-related atrophy. The shrinkage of the thymus with aging causes a decline in tissue mass and leads to certain structural changes [2, 7]. Interestingly, the most of the top ten upregulated and downregulated DEGs were ncRNA and microRNA at the 27^th^ week. A lot of research experiments have showed that the developmental processes of organs are regulated by both protein-coding and noncoding regions of the genome [36, 37]. Many transcripts of both ncRNA and microRNA such as lncRNA205 and MiR-205 contribute to thymopoiesis and postnatal thymus development [2, 38]. Thymic involution is strongly mediated by microRNA along with adipose tissue and cytokine alterations with the advancement in age [39]. The study of normal thymic structure and its alteration offers a cornerstone for better understanding of its immune function [40]. Since the structure and the function are closely related, the microstructure of different aged thymuses was explored by HE staining that showed a downtrend, except for a small increase at the 3^rd^ week, with significant alterations in ratio of the cortex to medulla. Similar changes in the aging thymus were also previously reported in rats [41–43], African ostrich chicks [44, 45], and cynomolgus macaques [46, 47]. In addition, we also found an accumulation of adipose tissue while aging. In a prior report, it was found that both adipocyte tissue and lipid-bearing cells increase within the thymic medulla with the advancement in age [48]. Thymic function during age-related atrophy is largely compromised of morphological alterations due to deposition of the adipose tissue within the immune organ together with the expansion of the perivascular spaces leading to greater risk of infections and autoimmune diseases [15, 49, 50]. Hence, all these studies proposed that the thymus of a developing chicken undergoes continued morphological alterations that must be driven by biomolecular and transcriptional dynamics in the immune organ microenvironment. Moreover, ncRNA and microRNA might be associated with age-associated thymic atrophy in the chicken at the 27^th^ week of age at the transcriptional level.

Based upon the consistency in morphological and transcriptional findings, we performed GO and KEGG enrichment analyses using the most relevant DEGs at the 27^th^ week of postnatal thymus development. In our study, DEGs annotated for GO analysis revealed the top three enriched molecular function categories as the binding activity, catalytic activity, and transporter activity. Several previous studies have reported that the catalytic activity plays an important role in thymic mass reduction [51–53] along with the transport and binding of fatty acids at adipose tissue deposition sites during age-related thymus atrophy [54, 55]. According to bioinformatics analysis, cell cycle, DNA replication, cytokine-cytokine receptor, and mismatch repair pathways were significantly enriched in the KEGG pathways in the chicken thymus at the 27^th^ week of age. Almost similar transcripts participate in cell cycle regulation as implicated in DNA replication during transition of the G2-to-M or S phase [56, 57]. Cytokines mainly regulate immune response [58]. The balance between proinflammatory and anti-inflammatory cytokines is important for the body to maintain normal function [59, 60]. A previous study has shown that age-related thymic atrophy is associated with decreased expression of interleukin-7 (IL-7) [61]. Cyclin-A2 (CCNA2), cyclin-B2 (CCNB2), and cyclin-dependent kinase-1 (CDK1) genes showed similar expression patterns in RNA-seq and q-PCR results at the 27^th^ week of age. CCNA2 is implicated in cell division and may exert antiproliferative effects and cell cycle arrest in the mammalian spleen during immunosuppression [62]. CDKs regulate the cell cycle functions and are involved in differentiation, transcription, and mRNA processing in eukaryotic cells [57, 63]. In agreement with our results, a similar RNA-seq study in murine found that several members of the cyclin family including CCNA2 and CCNB2 as well as CDKs are strongly associated with cell cycle regulation during the early stages of age-related thymic involution [64]. Thus, we concluded that the decrease in proliferation in the chicken thymus might be attributed to the arrest of the cell cycle, especially at the G1/S checkpoint by cyclin and cyclin-dependent kinases during age-associated thymic atrophy in chickens at the 27^th^ week of age.

## 5. Conclusion

The current study revealed that during postnatal development, the thymus undergoes severe atrophy. The thymus structure was damaged and gene expression changed dramatically, especially at the 27^th^ week of age. Moreover, we found significant activation of several signaling pathways such as the cytokine-cytokine receptor interaction and cell cycle signaling pathways. Hence, it may be inferred that those signaling pathways might be closely related to the postnatal chicken thymus development.

## Figures and Tables

**Table 1:** Primers used for real-time PCR.

Gene	Primer sequences (5′ to 3′)	Accession no.
*β*-Actin	f-TTGTTGACAATGGCTCCGGT	NM_205518.1
	r-TCTGGGCTTCATCACCAACG	
PCNA	f- TCTGAGGGCTTCGACACCTA	NM_204170.2
	r- AACCTTTTCCTGATTTGGTGCTT	
CDK1	f-TCTTCTGCCATTCAAGACGAGTTCTG	NM_205314.1
	r-GATCCTAGCAGTACCTCTGGAGACC	
CCNA2	f-GCCTTGCCTCATGGACCTTCAC	NM_205244.1
	r-TCTGGTGCGTCAATAAGCGATACTG	
CCNB2	f-TCCAGGTCCACTCAAGGTTCCAG	NM_001004369.1
	r-CCACCAACTGAAGCCTCTTACGAG	

## Data Availability

RNA-seq data supporting the current study has been deposited in the National Center for Biotechnology Information (NCBI) Sequence Read Archive (SRA) under accession code PRJNA492316. The SRA data will be accessible with the following link after publication of the article: https://www.ncbi.nlm.nih.gov/sra/PRJNA492316, or before by contacting the corresponding author.

## Supplementary Materials

Supplementary Materials Venn diagrams showing downregulated (a) and upregulated (b) DGEs at different time points compared with the 0 week. H1, H2, and H3 denote three biological replicates.
